# Endothelial Progenitor Cells and Vascular Alterations in Alzheimer’s Disease

**DOI:** 10.3389/fnagi.2021.811210

**Published:** 2022-01-26

**Authors:** Antía Custodia, Alberto Ouro, Daniel Romaus-Sanjurjo, Juan Manuel Pías-Peleteiro, Helga E. de Vries, José Castillo, Tomás Sobrino

**Affiliations:** ^1^NeuroAging Group (NEURAL), Clinical Neurosciences Research Laboratory (LINC), Health Research Institute of Santiago de Compostela (IDIS), Santiago de Compostela, Spain; ^2^Neuroimmunology Research Group, Department of Molecular Cell Biology and Immunology, Amsterdam Neuroscience, Amsterdam UMC, Amsterdam, Netherlands; ^3^Neuroimaging and Biotechnology Laboratory (NOBEL), Clinical Neurosciences Research Laboratory (LINC), Health Research Institute of Santiago de Compostela (IDIS), Santiago de Compostela, Spain

**Keywords:** Alzheimer’s disease, biomarkers, blood brain barrier dysfunction, endothelial progenitor cells, endothelial repair, neurotoxicity, two-hit vascular hypothesis, vascular alteration

## Abstract

Alzheimer’s disease (AD) is a neurodegenerative disease representing the most common type of dementia worldwide. The early diagnosis of AD is very difficult to achieve due to its complexity and the practically unknown etiology. Therefore, this is one of the greatest challenges in the field in order to develop an accurate therapy. Within the different etiological hypotheses proposed for AD, we will focus on the two-hit vascular hypothesis and vascular alterations occurring in the disease. According to this hypothesis, the accumulation of β-amyloid protein in the brain starts as a consequence of damage in the cerebral vasculature. Given that there are several vascular and angiogenic alterations in AD, and that endothelial progenitor cells (EPCs) play a key role in endothelial repair processes, the study of EPCs in AD may be relevant to the disease etiology and perhaps a biomarker and/or therapeutic target. This review focuses on the involvement of endothelial dysfunction in the onset and progression of AD with special emphasis on EPCs as a biomarker and potential therapeutic target.

## Introduction

Alzheimer’s disease (AD) is the main neurodegenerative disease leading to dementia and cognitive impairment. According to *World Alzheimer Report 2019: Attitudes to dementia* there are 50 million people with dementia (two-thirds with AD), with an expected increase of more than 152 million patients by the year 2050 (International, 2019).

Alzheimer’s disease can be classified according to its onset. Early-onset AD, which is mostly caused by autosomal dominant mutations; and late-onset AD, which accounts for most cases and whose etiology remains unclear. The most studied mutations responsible for autosomal dominant AD occur in the β-amyloid (Aβ) precursor protein (APP), presenilin 1 (PS1), and presenilin 2 (PS2) genes. However, those mutations collectively represent less than 1% of total cases. Late-onset AD is diagnosed from the age of 65 onward, and it has a multifactorial cause in which both environmental and genetic risk factors are involved (Giri et al., 2017). Among, different vascular-associated genetic risk factors, those corresponding to ε4 allele of *APOE (APOE* ε*4*), *phatidylinositol binding clathrin assembly protein* (*PICALM*), *clusterin* (*CLU*) or *sortilin related receptor-1* (*SORL1*) genes have been identified in AD (Sweeney et al., 2019). From all of them, *APOE*ε*4* is the most studied. Curiously, all affect Aβ clearance across the blood-brain barrier (BBB) (Sweeney et al., 2019).

Due to the symptomatic complexity of the disease and its similarity with other types of dementia, an accurate *premortem* diagnosis of AD is particularly challenging. Regrettably, the definitive diagnosis is made by *postmortem* brain tissue histological tests. Currently, techniques such as magnetic resonance imaging (MRI) (Wolz et al., 2011; Bron et al., 2017), positron emission tomography (PET) (Salmon et al., 1994; Ding et al., 2019), and detection of biomarkers released into the cerebrospinal fluid (CSF) (Blennow et al., 2001; Khoonsari et al., 2019), are only able to detect AD patients in late stages of the disease. Furthermore, different techniques have been recently developed to detect mild cognitive impairment (MCI) biomarkers (a stage prior to AD), including the analysis of hippocampal BBB leakage by dynamic contrast-enhanced MRI K_*trans*_ values (Montagne et al., 2015, 2020; Nation et al., 2019) and the analysis of platelet-derived growth factor receptor-β (sPDGFRβ) levels in CSF, a marker of pericyte damage (Montagne et al., 2015; Nation et al., 2019). However, none of these techniques became the gold standard in clinical practice for the early diagnosis of AD.

Currently, there are different hypotheses about the AD onset:

(a) β-amyloid deposit and hyperphosphorylated tau protein hypothesis: AD is originated from the presence of extraneuronal amyloid plaques formed by amyloid fibers composed of Aβ protein, and intraneuronal neurofibrillary tangles (NFTs), that are mainly formed by paired helical filaments (PHF) of the hyperphosphorylated tau protein (Gallardo and Holtzman, 2019; Paroni et al., 2019; Arnsten et al., 2021).
(b) Cholinergic hypothesis: the cause of AD is due to alterations of the cholinergic system. In AD there are modifications in cholinergic transport, acetylcholine release, expression of cholinergic receptors, reduction of acetylcholine transferase activity, and loss of cholinergic neurons. These events are relevant for AD since the cholinergic system is closely related to memory (Mufson et al., 2008; Hampel et al., 2018).
(c) Two-hit vascular hypothesis: a damage in cerebral vasculature (hit one) induces the accumulation of Aβ in the brain (hit two) (Zlokovic, 2011). (See below section “Two-hit vascular hypothesis” for more information.) Several studies support the early appearance of vascular alterations in AD (Sweeney et al., 2018; Apátiga-Pérez et al., 2021; Hussain et al., 2021; Kurz et al., 2021). In this scenario, endothelial progenitor cells (EPCs) appear as a possible therapeutic target by considering their involvement in the maintenance of vasculature.

For all the aforementioned, this mini-review is focused on vascular and angiogenic alterations in AD, besides the potential key role of EPCs on the AD’s etiology, and their potential as a therapeutic biomarker.

## Evidence of Vascular Alterations in Alzheimer’s Disease

**FIGURE 1:**
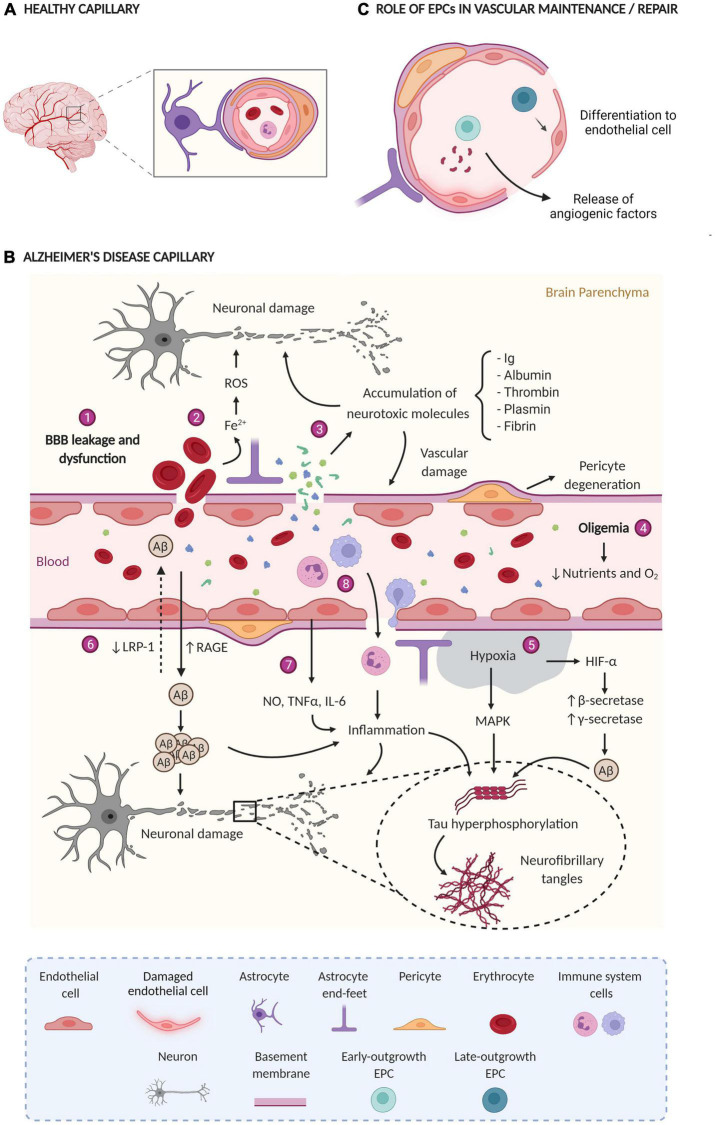
**(A)** Healthy capillary; the BBB has tight junctions between endothelial cells, pericytes enveloping endothelial cells, astrocytes, and normal blood flow. **(B)** AD capillary; Main vascular alterations occurring in AD: (1) the rupture and dysfunction of the BBB increase the permeability of different cells and molecules; (2) the accumulation of erythrocytes in the parenchyma generates neurotoxic products (Fe^2+^) that damage brain cells through the production of ROS; (3) the extravasation and consequent accumulation of neurotoxic molecules induces neurodegeneration and vascular damage; (4) oligemia induces a reduction in nutrient and oxygen supply, (5) generating hypoxic zones increases Aβ production and tau phosphorylation; (6) Aβ flow through the BBB is impaired due to a lower expression of LRP-1 and a higher expression of RAGE, leading to Aβ accumulation in the brain parenchyma; (7) blood vessels release a large amount of proinflammatory mediators (NO, TNFα, and IL-6) that together with (8) the extravasation of immune system cells generate inflammation. All together finally generate neuronal damage. ROS, reactive oxygen species; Ig, immunoglobulins; BBB, blood-brain barrier; Aβ, amyloid β protein; LRP-1, low-density lipoprotein receptor-related protein 1, RAGE, receptor for advanced glycation end products; NO, nitric oxide; TNF-α, tumor necrosis factor-alpha; IL-6, interleukin-6, HIF-1α, hypoxia-induced factor 1α; MAPK, mitogen-activated protein kinase. **(C)** Role of EPCs in vascular maintenance/repair e-EPCs participate in vasculogenesis and/or angiogenesis in a paracrine manner through the release of proangiogenic factors. L-EPCs participate by directly differentiating into mature endothelial cells. Created with BioRender.com.

The alteration and dysfunction of the cerebral vasculature is an important component of AD pathophysiology (Figure 1A shows a healthy capillary and Figure 1B shows an AD capillary). Hence, this process could contribute to the appearance and progression of the disease as well as promote neurodegeneration, inflammation, Aβ accumulation, and tau phosphorylation (Sagare et al., 2012). Several findings acquired by neuroimaging techniques, analysis of *postmortem* brain samples, and CSF biomarkers detection support a vascular dysfunction in AD (Montagne et al., 2017; Sweeney et al., 2018).

Neuroimaging studies using ^18^F-fluorodeoxyglucose (FDG)-PET linked alterations in glucose transport to MCI and early AD. This was determined through a reduction in glucose transporter-1 (GLUT-1) in the BBB (Minoshima et al., 1997; Bailly et al., 2015; Nelson et al., 2016). P-glycoprotein (P-gp) is a membrane protein found in the endothelial cells of the BBB. Of interest, its principal function is to eliminate xenobiotics from cerebral parenchyma to the blood, although, it can also transport Aβ (Gil-Martins et al., 2020). Importantly, ^11^C-verapamil-PET studies described the dysfunction of P-gp in AD and MCI patients (Van Assema et al., 2012; Deo et al., 2014). Additionally, MRI approaches showed that early AD patients present BBB leakage in the gray matter and cortex, which is associated with cognitive impairment and a decreased cerebral blood flow in gray matter (Van De Haar et al., 2016a,b). Interestingly, the hippocampal BBB leakage occurs before suffering brain atrophy or dementia in MCI patients and APOEε4 carriers, in addition, it is regardless of Aβ and tau changes (Montagne et al., 2015, 2020; Nation et al., 2019). MRI also revealed the presence of microbleeds in the central nervous system in 45–78 and 25% of patients at the initial stages of AD (before developing dementia) or with MCI, respectively (Brundel et al., 2012; Uetani et al., 2013; Yates et al., 2014). These results agree with the hypothesis of an early endothelial dysfunction underlying the onset of AD.

Blood-brain barrier permeability was also determined in *postmortem* tissue (Figure 1B), where the leakage of certain substances (e.g., fibrinogen, fibrin, thrombin, plasmin, albumin, or immunoglobulins) from capillaries and the subsequent accumulation in the parenchyma was detected (Wisniewski and Kozlowski, 1982; Grammas et al., 2006; Ryu and McLarnon, 2009; Sengillo et al., 2013). Additionally, the extravasation of erythrocytes, peripheral macrophages, and neutrophils has been observed (Fiala et al., 2002; Cullen et al., 2005; Zenaro et al., 2015). Electron microscopy and immunostaining techniques have determined the degeneration of pericytes, considered key cells in the maintenance of the BBB (Baloyannis and Baloyannis, 2012; Sengillo et al., 2013). In this regard, an increase in sPDGFRβ levels, a marker of pericyte damage, was found in the CSF of both AD and MCI patients (Montagne et al., 2015; Miners et al., 2019; Nation et al., 2019). Elevated levels of sPDGFRβ correlate with the CSF/plasma albumin ratio and the levels of fibrinogen in CSF, both are markers of BBB leakage (Miners et al., 2019; Nation et al., 2019). In this sense, the existence of endothelial degeneration in AD patients and reduction in the length of the capillaries has been confirmed, as well as the reduced expression of tight junction proteins in capillaries (Baloyannis and Baloyannis, 2012; Halliday et al., 2016). Remarkably, several molecular changes have been also observed in the cerebral endothelium of AD patients, such as low expression of low-density lipoprotein receptor-related protein 1 (LRP-1) and GLUT-1 (Mooradian et al., 1997; Donahue et al., 2006), which validate previous neuroimaging studies. Curiously, LRP-1 removes Aβ from the brain in conjunction with P-gp (Storck et al., 2018). Moreover, other molecular changes including increased levels of the receptor for advanced glycation end products (RAGE) have been reported (Donahue et al., 2006). Interestingly, RAGE induces the uptake of circulatory Aβ to the cerebral parenchyma (Donahue et al., 2006). Cyclophilin A (CypA) and matrix metalloproteinase-9 (MMP-9) are also overexpressed, leading to the degradation of the BBB tight junctions (Halliday et al., 2016). *APOE*ε*4* increases the damage of the BBB by the activation of the cyclophilin-A-MMP-9 pathway (Bell et al., 2012; Halliday et al., 2013). Accordingly, APOEε*4* carriers have elevated levels of CypA, MMP9, and sPDGFRβ in the CSF (Montagne et al., 2020). In microvessels isolated from AD brains higher levels of inflammatory mediators were determined, such as nitric oxide (NO), tumor necrosis factor-alpha (TNF-α), and interleukin-6, and 8 (IL-6 and IL-8), MMPs, prostaglandins, and leukocyte adhesion molecules, compared with healthy controls (Dorheim et al., 1994; Grammas and Ovase, 2001; Thirumangalakudi et al., 2006). Interestingly, some of these molecules are implicated in angiogenesis (Grammas and Ovase, 2001; Grammas et al., 2006).

Alterations in angiogenesis also occur in AD (Steinman et al., 2021). Vascular dysregulation leads to a lack of oxygen in the brain (hypoxia), which eventually induces an up-regulation of pro-angiogenic proteins in brain vessels, such as vascular endothelial growth factor (VEGF), thrombin, or hypoxia-induced factor 1α (HIF-1α), among others (Grammas et al., 2006; Thirumangalakudi et al., 2006). Despite the increase in pro-angiogenic factors, there is no evidence of increased vascularization. Indeed, it has been shown that vascular density decreases (Baloyannis and Baloyannis, 2012). Although the causes underlying abnormal angiogenesis are not clear, *in vitro* and *in vivo* studies revealed that the Aβ peptide has anti-angiogenic effects (Paris et al., 2004). Accordingly, brain endothelial cells from AD patients have low levels of vascular-restricted mesenchyme homeobox 2 gene (*MEOX-2*). *MEOX-2* acts as a regulator for the proliferation of vascular cells. Low levels of the mentioned gene in AD generate aberrant angiogenesis that results in a decrease in cerebral microcirculation. In animal models, *MEOX-2* deletion reduces LRP-1 levels, leading to decreased Aβ efflux from the brain parenchyma to the blood, among other vascular alterations (Wu et al., 2005). Importantly, a hypoxic environment suppresses the expression of *MEOX-2* (Xia S. et al., 2012).

In summary, there is a large amount of evidence, from multiple approaches, that support the existence of a neurovascular component in the onset of AD. Remarkably, this vascular dysfunction starts before developing atrophy and/or dementia and continuous in later stages. Based on this evidence, the two-hit vascular hypothesis was proposed (Zlokovic, 2011).

## Two-Hit Vascular Hypothesis

Zlokovic (2005) suggested that neurovascular dysfunction contributes to the cognitive decline and neurodegeneration associated to AD. Later, his group proposed the two-hit vascular hypothesis, where damage in cerebral vasculature (hit one) induces the accumulation of Aβ in the brain (hit two) (Zlokovic, 2011).

The damage in the cerebral vasculature (hit one) can be caused by either several vascular risk factors (such as hypertension, diabetes, hypercholesterolemia, or smoking, among others), or by genetic risk factors like *APOE*ε*4*. The cerebral vasculature undergoes several outcomes during the injury (Figure 1B). Accordingly, the cerebral blood flow is reduced (oligemia), leading to hypoxia in some areas, and the subsequent release of reactive oxygen species (ROS) that promote cellular damage by oxidative stress (Carvalho et al., 2009), as well as inducing the expression of HIF-1α. In addition, HIF-1α. increases the expression and activity of β-secretase and the activity of γ-secretase. Therefore, this raises the amyloidogenic pathway and ultimately Aβ production (Zhang et al., 2007; Li et al., 2009). There is also a dysfunction in the BBB, leading to an increase in the permeability of toxic molecules and their accumulation in the brain parenchyma (mentioned in the previous section). Some of these molecules cause neurodegeneration and further increase the damage to the cerebral vasculature (Chen and Strickland, 1997; Mhatre et al., 2004; Paul et al., 2007; Chen et al., 2010). Moreover, the presence of erythrocytes in the parenchyma generates neurotoxic products, such as iron, leading to an increase of ROS and therefore, brain damage (Regan and Guo, 1998).

Finally, the cerebrovascular dysfunction triggers inflammation, defective Aβ clearance in the brain, and increased influx of peripheral Aβ through BBB (Figure 1B). Likewise, tau hyperphosphorylation is also promoted by these harmful processes leading to the formation of NFTs (Busciglio et al., 1995; Gordon-Krajcer et al., 2007; Fang et al., 2010; Koike et al., 2010; Lee D. et al., 2010). All these events result in the accumulation of Aβ and tau in the brain tissue and Aβ around cerebral blood vessels (hit two) (Sagare et al., 2012; Nelson et al., 2016; Sweeney et al., 2018). In fact, it has recently been described that insoluble vascular amyloid deposits could induce BBB disruption (Soto-Rojas et al., 2021). In summary, the two-hit vascular hypothesis suggests that the accumulation and hyperphosphorylation of tau are secondary to a vascular lesion and/or a lesion generated by Aβ (Grammas, 2011; Zlokovic, 2011). Moreover, there is a feedback between the cerebrovascular dysfunction and inflammation, since each one can induce the other (Clapp et al., 2004; Theofilis et al., 2021). Indeed, one of the risk factors for AD is chronic inflammation (Tao et al., 2018).

Overall, these events promote synaptic dysfunction, neuronal damage, neurodegenerative change, and finally neuronal death, thus generating dementia (Chen and Strickland, 1997; Regan and Guo, 1998; Grammas et al., 2000; Mhatre et al., 2004; Carvalho et al., 2009; Chen et al., 2010; Feuillette et al., 2010; Tian et al., 2013; Pereira et al., 2021).

## Endothelial Progenitor Cells and Their Potential Role in Alzheimer’s Disease

Since angiogenesis and the integrity of the BBB are both crucial for the development of AD, and EPCs are essentials in endothelial repair processes, it is tempting to propose that EPCs may have a key role in this disease. In recent years, our group has described that EPCs are involved in other neurovascular diseases such as stroke and migraine (Sobrino et al., 2007, 2011, 2012a,b; Rodríguez-Osorio et al., 2012; Rodríguez et al., 2016; Pías-Peleteiro et al., 2017).

Endothelial progenitor cells constitute a subtype of cells present in the blood that derive mostly from the bone marrow. These cells exhibit characteristics of both endothelial and stem cells since they can differentiate into mature endothelial cells and self-renew, respectively (Hristov et al., 2003; Yoder, 2012). Three surface markers are characteristics of EPCs: CD34, VEGF receptor 2 (VEGFR-2), and CD133. CD34 is a marker expressed by hematopoietic stem cells and certain types of mature endothelial cells. VEGFR-2 is a specific receptor of VEGF and is expressed in endothelial cells and uncommitted stem cells. CD133 is an early marker of hematopoietic stem cells (Hristov et al., 2003). EPCs participate in the maintenance of the endothelium by acting as a cellular reservoir for the replacement of dysfunctional endothelial cells or by releasing angiogenic growth factors (Figure 1C; Hristov et al., 2003; Lee et al., 2009; Malinovskaya et al., 2016). There are two different types of EPCs: (a) early-outgrowth EPCs (e-EPCs), circulatory angiogenic cells, or colony-forming unit endothelial cells (CFU-EC) which participate in both the process of network formation and the repair of injured endothelial cells in a paracrine way by secreting different angiogenic factors; and (b) late-outgrowth EPCs (l-EPCs), endothelial outgrowth cells, or endothelial colony-forming cells, which improve angiogenesis by differentiating into mature endothelial cells (Hur et al., 2004; Bauman et al., 2018). In addition to their functionality, both types of EPCs can be recognized by characterization *in vitro.* Whereas e-EPCs appear after a few days in culture and form colonies with spindle-shaped cells around them, l-EPCs appear after 2–3 weeks in culture and present a cobblestone shape (Hur et al., 2004).

The number of EPCs has been proposed as a possible surrogate marker of vascular function, and low EPCs counts are associated with higher cardiovascular risk (Hill et al., 2009; Bitterli et al., 2016; Hayek et al., 2016). These cells have also been implicated in the maintenance of cerebral endothelial vasoreactivity in healthy subjects (Chung et al., 2015). EPCs may have an important role in different nervous system diseases. For example, EPCs attach to the endothelium and promote the formation of new vessels after an ischemia and/or hypoxia event. Consequently, EPCs induce and modulate vasculogenesis and angiogenesis in those hypoxic areas, as well as stimulate re-endothelialization of injured vessels (Yoder, 2012). Indeed, high EPCs levels have been associated with a good functional and neurological prognosis, besides a reduction of the infarct growth in patients with ischemic stroke (Sobrino et al., 2007). In addition, the EPCs percentage in blood was associated with serum levels of VEGF, stromal cell-derived factor-1α and, active MMP-9 (Sobrino et al., 2012b). Interestingly, patients treated with statins had larger EPCs levels and, therefore, a better outcome (Sobrino et al., 2012a). This relationship has also been established in intracerebral hemorrhage (ICH) patients (Sobrino et al., 2011; Pías-Peleteiro et al., 2017). Notably, ICH patients who presented the Pro72 single-nucleotide polymorphism in the *tumoral protein 53* (*Tp53*) gene had higher levels of circulating EPCs, EPCs-mobilizing cytokines, and, eventually, better functional outcome (Rodríguez et al., 2016). These factors were related to greater neovascularization. Likewise, a reduced number of EPCs has been shown in patients with migraines, especially during attacks (Rodríguez-Osorio et al., 2012). Recently, a relationship has been observed between the elevated levels of EPCs and cerebral small vessel disease burden, which is a risk factor for the development of AD (Kapoor et al., 2021). Curiously, sickle cell anemia (SCA) patients, a monogenic disease that affect erythrocyte membranes, present a high risk to develop a small vessels disease such as ischemic, hemorrhagic, and silent strokes (Ito et al., 2020). Newly, genetic alterations in l-EPCs of genes involved in angiogenesis, coagulation, inflammation, apoptosis, and cell adhesion have been observed in SCA patients that suffered a stroke (Ito et al., 2020). Therefore, EPCs seem to be involved in cerebrovascular diseases.

Several studies have analyzed the number of circulating EPCs in AD patients and their ability to form CFU-EC colonies (Table 1). However, there are discrepancies in these results. Lee and co-workers determined that AD patients did not present significant differences in the number of circulating EPCs compared with subjects without AD who present cardiovascular risk factors. Despite that, AD patients had a significant reduction in CFU-EC colony formation, and this decrease was correlated with a greater cognitive impairment (Lee et al., 2009). In accordance with some of these findings, other works concluded that there are no significant differences in the number of circulating EPCs in AD patients and controls (Breining et al., 2016; Haiyuan et al., 2020). However, EPCs from moderate and severe AD showed functional alterations in culture, such as reduced adhesion and migration capacity, compared to mild AD and controls (Haiyuan et al., 2020). Conversely, a clinical study indicated that AD patients had a reduced number of circulating EPCs compared with control subjects and that a lower number of EPCs correlates with greater cognitive impairment (Kong et al., 2011). The discrepancies in these studies may be due to the age of the subjects since the number of circulating EPCs decrease with age (Jie et al., 2009; Xia W. H. et al., 2012). In this regard, Breining et al. (2016) and Haiyuan et al. (2020) analyzed data from older subjects than Lee et al. (2009) and Kong et al. (2011). As a result, the physiological decrease in the number of EPCs with aging may mask the results. Furthermore, the difference in the results could also be due to the different inclusion and exclusion criteria used in the studies besides the likely existence of underlying diseases. Moreover, none of the studies differentiated between APOEε4 carriers, and both APOEε4 carriers and low levels of EPCs are considered cardiovascular risk factors (Hill et al., 2009; Mahley, 2016). Although the number of circulating EPCs is controversial, several studies reported functional alterations in EPCs (Lee et al., 2009; Haiyuan et al., 2020). In concordance with these results, another study in AD patients observed that e-EPCs presented reduced chemotaxis, and paracrine angiogenic properties, increased senescence; and altered gene expression (most of them related to physiological cellular processes) (Lee S. T. et al., 2010). Other factors involved in the pathogenesis of AD such as Aβ_1–42_ accumulation may also influence the functionality of e-EPCs. High concentrations of Aβ_1–42_ induce apoptosis, with AD-derived e-EPCs being more susceptible (Lee S. T. et al., 2010). Moreover, aging leads to functional alterations in e-EPCs that correlate with endothelial dysfunction (Heiss et al., 2005). Therefore, although the results are compromised, all the aforementioned studies analyzing EPCs in culture observe functional alterations of these cells in AD regardless of their circulating number.

**TABLE 1:** Summary of relevant preclinical and clinical studies on association between EPCs and AD.

Endothelial Progenitor Cells And Alzheimer’s Disease			
**Preclinical studies**			

**References**	**Study**	**Animal model**	**Results**

Safar et al., 2016	Injection of e-EPCs in the tail vein of SCO-induced AD-like pathological rat model.	Induced model	EPCs (approximately 1 × 10^6^ cells) administration induced: improvement in learning and memory measured by Morris water Maze test; attenuation of amyloid plaque deposition detected by histology; suppression of Aβ and p-tau levels determined by ELISA; and reversal of neurotransmitter aberrations analyzed by ELISA.
Yuan et al., 2016	Injection of l-EPCs in the tail vein of APP/PS1 transgenic mice.	Transgenic model	Enhanced penetration of exogenous EPCs into the brain of APP/PS1 transgenic mouse model of AD in comparison with controls.
Zhang et al., 2018	Injection of l-EPCs in the hippocampus of APP/PS1 transgenic mice.	Transgenic model	EPCs (approximately 4 × 10^5^ cells) administration into the hippocampus induced: up-regulation of tight junction proteins (ZO-1, CLN-5, and occludin) measured by immunofluorescence and western blot; increment of microvessel density showed by immunofluorescence of CD31; angiogenesis in the hippocampus and cortex described by immunofluorescence of CD31; anti-apoptotic effect measured by western blot; reduction of area and intensity of Aβ plaques in the hippocampus analyzed by immunohistochemistry; and improvement in memory and learning measured by Morris water maze.

**Clinical studies**			

**References**	**Study**	**Sample**	**Results**

Maler et al., 2006	Relation between the number of CD34^+^ cells, CSF AB levels, and early AD	Venous blood and CSF	Significantly decreased CD34^+^ cells in early AD, levels of these cells were inversely correlated with significantly inverse correlation between the number of CD34^+^ cells, Aβ_1–42_ levels, and Aβ_42/40_ ratio in CSF.
Lee et al., 2009	Relation between the number of EPCs and their ability to form colonies and AD.	Venous blood	No significant differences in the number of circulating EPCs between patients and control groups. Significantly reduction in CFU-EC colony formation in AD patients. Correlation between lower number of colonies and greater cognitive impairment.
Lee S. T. et al., 2010	Relation between e-EPCs functional characteristics and AD.	Venous blood	Significant reduction in chemotaxis and paracrine angiogenesis properties, increase in senescence and altered genes expression in AD e-EPCs. Induction of apoptosis and functional alterations in e-EPCs by high concentrations of Aβ_1–42_. Greater susceptibility in e-EPCs in AD than in controls with cardiovascular risk factors.
Stellos et al., 2010	Relation between the concentrations of circulating CD34^+^/CD133^+^ and CD34^+^ progenitor cells and AD.	Venous blood	Significantly increase in circulating CD34^+^/CD133^+^ and CD34^+^ progenitor cells in moderate-severe AD compared to controls. Significantly inverse correlation between the number of circulating CD34^+^/CD133^+^ and CD34^+^ progenitor cells, cognitive function, and age in AD patients.
Bigalke et al., 2011	Relation between adipocytokines and CD34^+^ progenitor cells in AD.	Venous blood	Statistically significant increase in circulating CD34^+^ progenitor cells and decrease in leptin plasma levels in early AD. Significantly inverse correlation between the number of circulating CD34^+^ progenitor cells and leptin plasma levels.
Kong et al., 2011	Relation between the number of EPCs and AD.	Venous blood	Decreased number of circulating EPCs in AD patients, statistically significant. Correlation between lower number of circulating EPCs and lower Mini-Mental State Examination score.
Breining et al., 2016	Relation between the number of EPCs and AD.	Venous blood	No significant differences in the number of circulating EPCs between AD patients and control groups.
Nation et al., 2018	Relation between number of circulating EPCs, CD133^+^/CD34^+^ and CD34^+^ cells and MCI, memory, posterior cortical thickness, and hippocampal perfusion.	Venous blood	Significantly decreased number of circulating EPCs, CD133^+^/CD34^+^ and CD34^+^ cells in MCI. Significant association between low levels of CD34^+^ cells, worse memory, lower posterior cingulate gyrus cortical thickness, and bilateral hippocampal hyperperfusion
Haiyuan et al., 2020	Relation between the number of EPCs, their adhesion and migration capacity, and AD.	Venous blood	No significant differences in the number of circulating EPCs between patients and control groups. Significant reduction in migration and adhesion properties in moderate and severe AD compared to mild AD and controls.

Circulating progenitor cells (CPCs) are cells involved in tissue maintenance and repair (Sidney et al., 2014). Different authors have described CD34 as a marker for this cell lineage (Sidney et al., 2014). Within CPCs, different subpopulations of cells can be determined not only by the different markers expressed on their plasma membrane but also by the ability to differentiate into one or more mature cell types. EPCs represent one of these subpopulations (Sidney et al., 2014). Therefore, we have included different studies relating CPCs and AD in this mini-review, which shows some discrepancies in the results (Table 1). For example, there is a significant increase in circulating CD34^+^/CD133^+^ and CD34^+^ progenitor cells in moderate-severe AD compared to healthy subjects (Stellos et al., 2010) and CD34^+^ cells in early AD (Bigalke et al., 2011). In contrast, another study reported a reduction in the levels of CD34^+^ cells in early AD, and the number of these cells was inversely correlated with Aβ_1–42_ levels and Aβ_42/40_ ratio in CSF (Maler et al., 2006). Moreover, the number of CD34^+^ and CD34^+^/CD133^+^ cells in AD patients was inversely correlated with cognitive function and age (Stellos et al., 2010). Contrary, another study found that in patients with coronary artery disease, lower numbers of CPCs are associated with cognitive impairment (Moazzami et al., 2020). Regarding cognitive impairment, patients with MCI present reduced levels of circulating EPCs as well as CD34^+^/CD133^+^ and CD34^+^ progenitor cells (Nation et al., 2018). Concerning CD34^+^ cells, a lower number of these cells was associated with worse memory, lower posterior cingulate gyrus cortical thickness, and bilateral hippocampal hyperperfusion (Nation et al., 2018). However, the reduced number of studies and their controversial results highlight the need of further studies in order to reach more conclusive results about EPCs and CPCs in the onset and progression of AD.

Additionally to the analysis of the number of EPCs in AD, the therapeutic potential of these cells has also been suggested in different animal models (Table 1). For instance, e-EPCs were injected intravenously into repeated scopolamine (SCO)-induced cognitive impairment rats, an experimental model that replicates biomarkers of AD (Safar et al., 2016). As a result, there was an improvement in learning and memory; besides attenuation of Aβ plaque deposition, suppression of Aβ and p-tau levels, and reversal of neurotransmitter aberrations. L-EPCs were also injected intravenously in APP/PS1 transgenic mice, exhibiting an enhanced penetration of exogenous EPCs into the brain compared to controls (Yuan et al., 2016). Subsequently, using the same transgenic mice model, l-EPCs were injected directly into the hippocampus (Zhang et al., 2018). The transplantation of EPCs up-regulated tight junction proteins (such as zonula occludens-1, occludin and, claudin-5) in the BBB, increasing microvessels density and promoting angiogenesis in the hippocampus and cortex. The EPCs also exerted an anti-apoptotic effect promoting neuronal survival in the hippocampus. In addition, a reduction in the area and intensity of Aβ plaques in the hippocampus and cerebral cortex was observed. Moreover, learning and memory were significantly improved in AD mice (APP/PS1) after EPCs transplantation (for more details see Table 1). Therefore, the use of transfected EPCs has been proposed as a possible treatment pathway in AD by taking advantage of their ability to home to the damaged BBB. Recently, transfected EPCs that release antibodies against Aβ and reduce its aggregation have been generated (Heller et al., 2020). However, this novel therapeutic approach has not yet been tested *in vivo*. Although, in other neurological pathologies, such as traumatic brain injury, the intraventricular administration of l-EPCs resulted in greater integrity of the BBB and increased angiogenesis in a mouse model (Huang et al., 2013). In ischemic stroke, intra-arterial administration of l-EPCs resulted in reduced infarct volume, as well as increased angiogenesis and vascular density (Lin et al., 2020). Therefore, EPCs are postulated as a good therapeutic option for pathologies that present BBB alterations.

## Discussion

Although AD is the main type of dementia worldwide, its etiology remains unclear. The Zlokovic two-hit vascular hypothesis proposes that AD starts from initial damage in the cerebral vasculature. Different studies in AD have demonstrated that there is a dysfunction of the BBB leading to hypoperfusion and hypoxia, accumulation of Aβ and hyperphosphorylation of tau, accumulation of neurotoxic molecules, and inflammation, among others in cerebral parenchyma. Altogether, these mechanisms cause neurodegeneration, neuronal dysfunction, an increase of pro-angiogenic molecules, and aberrant angiogenesis.

The basis of the decreased angiogenesis seen in AD remains unclear, although it has been proposed that it is due to either the accumulation of Aβ or the low expression of *MEOX-2*. However, the main cells responsible for carrying out angiogenesis in hypoxic sites are EPCs. Therefore, these cells could be involved in aberrant angiogenesis.

In the last years, clinical studies have analyzed the relationship between the number of EPCs and AD patients. The obtained data were contradictory since some studies did not observe significant differences in the number of EPCs, while others did. As discussed above, age and inclusion criteria may lead to observed differences. Interestingly, studies using animal models of AD showed that exogenous administration of EPCs improved learning, memory, and angiogenesis, attenuated Aβ deposition, reduced p-tau levels, and up-regulated the number of tight junctions, among others.

Taking all these data into account, we can conclude that there are several vascular and angiogenic alterations in AD and that EPCs may play a key role in endothelial and BBB dysfunction associated with AD. Moreover, *in vivo* studies using EPCs as a therapeutic approach open a possible new path for the treatment of AD. However, further studies are necessary to confirm the potential key role of EPCs as an early diagnostic and therapeutic biomarker in AD, and to elucidate the underlying mechanisms associated with the EPC’s therapeutic properties.

## Author Contributions

JC and TS: conceptualization. AC, AO, DR-S, and JP-P: bibliographic study. JC, HV, and TS: supervision. AC, AO, and DR-S: writing – original draft. All authors contributed to writing – review and editing.

## Conflict of Interest

The authors declare that the research was conducted in the absence of any commercial or financial relationships that could be construed as a potential conflict of interest.

## Publisher’s Note

All claims expressed in this article are solely those of the authors and do not necessarily represent those of their affiliated organizations, or those of the publisher, the editors and the reviewers. Any product that may be evaluated in this article, or claim that may be made by its manufacturer, is not guaranteed or endorsed by the publisher.

## References

[B1] Apátiga-Pérez R. Soto-Rojas L. O. Campa-Córdoba B. B. Luna-Viramontes N. I. Cuevas E. Villanueva-Fierro I. (2021). Neurovascular dysfunction and vascular amyloid accumulation as early events in Alzheimer’s disease. *Metab. Brain Dis.* 2021 1–12. 10.1007/S11011-021-00814-4 34406560

[B2] Arnsten A. F. T. Datta D. Del Tredici K. Braak H. (2021). Hypothesis: tau pathology is an initiating factor in sporadic Alzheimer’s disease. *Alzheimers Dement.* 17:115. 10.1002/ALZ.12192 33075193 PMC7983919

[B3] Bailly M. Destrieux C. Hommet C. Mondon K. Cottier J. P. Beaufils E. (2015). Precuneus and Cingulate Cortex Atrophy and Hypometabolism in Patients with Alzheimer’s Disease and Mild Cognitive Impairment: mRI and 18F-FDG PET Quantitative Analysis Using FreeSurfer. *Biomed Res. Int.* 2015:583931. 10.1155/2015/583931 26346648 PMC4539420

[B4] Baloyannis S. Baloyannis I. (2012). The vascular factor in Alzheimer’s disease: a study in Golgi technique and electron microscopy. *J. Neurol. Sci.* 322 117–121. 10.1016/J.JNS.2012.07.010 22857991

[B5] Bauman E. Granja P. Barrias C. (2018). Fetal bovine serum-free culture of endothelial progenitor cells-progress and challenges. *J. Tissue Eng. Regen. Med.* 12 1567–1578. 10.1002/TERM.2678 29701896

[B6] Bell R. Winkler E. Singh I. Sagare A. Deane R. Wu Z. (2012). Apolipoprotein E controls cerebrovascular integrity via cyclophilin A. *Nature* 485 512–516. 10.1038/NATURE11087 22622580 PMC4047116

[B7] Bigalke B. Schreitmüller B. Sopova K. Paul A. Stransky E. Gawaz M. (2011). Adipocytokines and CD34+ Progenitor Cells in Alzheimer’s Disease. *PLoS One* 6:e20286. 10.1371/JOURNAL.PONE.0020286 21633502 PMC3102092

[B8] Bitterli L. Afan S. Bühler S. Disanto S. Zwahlen M. Schmidlin K. (2016). Endothelial progenitor cells as a biological marker of peripheral artery disease. *Vasc. Med.* 21 3–11. 10.1177/1358863X15611225 26511986

[B9] Blennow K. Vanmechelen E. Hampel H. (2001). CSF total tau, Aβ42 and phosphorylated tau protein as biomarkers for Alzheimer’s disease. *Mol. Neurobiol.* 24 87–97. 10.1385/MN:24:1-3:087 11831556

[B10] Breining A. Silvestre J. S. Dieudonné B. Vilar J. Faucounau V. Verny M. (2016). Biomarkers of vascular dysfunction and cognitive decline in patients with Alzheimer’s disease: no evidence for association in elderly subjects. *Aging Clin. Exp. Res.* 28 1133–1141. 10.1007/s40520-016-0535-4 26803509

[B11] Bron E. E. Smits M. Papma J. M. Steketee R. M. E. Meijboom R. de Groot M. (2017). Multiparametric computer-aided differential diagnosis of Alzheimer’s disease and frontotemporal dementia using structural and advanced MRI. *Eur. Radiol.* 27 3372–3382. 10.1007/s00330-016-4691-x 27986990 PMC5491625

[B12] Brundel M. Heringa S. de Bresser J. Koek H. Zwanenburg J. Jaap Kappelle L. (2012). High prevalence of cerebral microbleeds at 7Tesla MRI in patients with early Alzheimer’s disease. *J. Alzheimers. Dis.* 31 259–263. 10.3233/JAD-2012-120364 22531417

[B13] Busciglio J. Lorenzo A. Yeh J. Yankner B. A. (1995). β-Amyloid fibrils induce tau phosphorylation and loss of microtubule binding. *Neuron* 14 879–888. 10.1016/0896-6273(95)90232-5 7718249

[B14] Carvalho C. Correia S. C. Santos R. X. Cardoso S. Moreira P. I. Clark T. A. (2009). Role of mitochondrial-mediated signaling pathways in Alzheimer disease and hypoxia. *J. Bioenerg. Biomembr.* 41 433–440. 10.1007/s10863-009-9247-1 19830532 PMC4054815

[B15] Chen B. Cheng Q. Yang K. Lyden P. D. (2010). Thrombin mediates severe neurovascular injury during ischemia. *Stroke* 41 2348–2352. 10.1161/STROKEAHA.110.584920 20705928

[B16] Chen Z. L. Strickland S. (1997). Neuronal death in the hippocampus is promoted by plasmin-catalyzed degradation of laminin. *Cell* 91 917–925. 10.1016/S0092-8674(00)80483-3 9428515

[B17] Chung C. P. Huang P. H. Chen J. S. Chen J. W. Yang K. Y. (2015). The level of circulating endothelial progenitor cell is associated with cerebral vasoreactivity: a pilot study. *Biomed Res. Int.* 2015:258279. 10.1155/2015/258279 26550564 PMC4624880

[B18] Clapp B. R. Hingorani A. D. Kharbanda R. K. Mohamed-Ali V. Stephens J. W. Vallance P. (2004). Inflammation-induced endothelial dysfunction involves reduced nitric oxide bioavailability and increased oxidant stress. *Cardiovasc. Res.* 64 172–178. 10.1016/J.CARDIORES.2004.06.020/2/64-1-172-FIG6.GIF 15364625

[B19] Cullen K. M. Kócsi Z. Stone J. (2005). Pericapillary haem-rich deposits: evidence for microhaemorrhages in aging human cerebral cortex. *J. Cereb. Blood Flow Metab.* 25 1656–1667. 10.1038/sj.jcbfm.9600155 15917745

[B20] Deo A. K. Borson S. Link J. M. Domino K. Eary J. F. Ke B. (2014). Activity of P-glycoprotein, a β-amyloid transporter at the blood-brain barrier, is compromised in patients with mild Alzheimer disease. *J. Nucl. Med.* 55 1106–1111. 10.2967/jnumed.113.130161 24842892 PMC4691246

[B21] Ding Y. Sohn J. Kawczynski M. Trivedi H. Harnish R. Jenkins N. (2019). A Deep Learning Model to Predict a Diagnosis of Alzheimer Disease by Using 18 F-FDG PET of the Brain. *Radiology* 290 456–464. 10.1148/RADIOL.2018180958 30398430 PMC6358051

[B22] Donahue J. E. Flaherty S. L. Johanson C. E. Duncan J. A. Silverberg G. D. Miller M. C. (2006). RAGE, LRP-1, and amyloid-beta protein in Alzheimer’s disease. *Acta Neuropathol.* 112 405–415. 10.1007/s00401-006-0115-3 16865397

[B23] Dorheim M. Tracey W. Pollock J. Grammas P. (1994). Nitric oxide synthase activity is elevated in brain microvessels in Alzheimer’s disease. *Biochem. Biophys. Res. Commun.* 205 659–665. 10.1006/BBRC.1994.2716 7528015

[B24] Fang H. Zhang L. Meng F. Du X. Zhou J. (2010). Acute hypoxia promote the phosphorylation of tau via ERK pathway. *Neurosci. Lett.* 474 173–177. 10.1016/J.NEULET.2010.03.037 20304032

[B25] Feuillette S. Miguel L. Frébourg T. Campion D. Lecourtois M. (2010). Drosophila models of human tauopathies indicate that Tau protein toxicity in vivo is mediated by soluble cytosolic phosphorylated forms of the protein. *J. Neurochem.* 113 895–903. 10.1111/J.1471-4159.2010.06663.X 20193038

[B26] Fiala M. Liu Q. Sayre J. Pop V. Brahmandam V. Graves M. (2002). Cyclooxygenase-2-positive macrophages infiltrate the Alzheimer’s disease brain and damage the blood-brain barrier. *Eur. J. Clin. Invest.* 32 360–371. 10.1046/J.1365-2362.2002.00994.X 12027877

[B27] Gallardo G. Holtzman D. M. (2019). Amyloid-β and Tau at the Crossroads of Alzheimer’s Disease. *Adv. Exp. Med. Biol.* 1184 187–203. 10.1007/978-981-32-9358-8_16 32096039

[B28] Gil-Martins E. Barbosa D. J. Silva V. Remião F. Silva R. (2020). Dysfunction of ABC transporters at the blood-brain barrier: role in neurological disorders. *Pharmacol. Ther.* 213:107554. 10.1016/J.PHARMTHERA.2020.107554 32320731

[B29] Giri M. Shah A. Upreti B. Rai J. C. (2017). Unraveling the genes implicated in Alzheimer’s disease (Review). *Biomed. Reports* 7 105–114. 10.3892/br.2017.927 28781776 PMC5526178

[B30] Gordon-Krajcer W. Kozniewska E. Lazarewicz J. W. Ksiezak-Reding H. (2007). Differential changes in phosphorylation of tau at PHF-1 and 12E8 epitopes during brain ischemia and reperfusion in gerbils. *Neurochem. Res.* 32 729–737. 10.1007/s11064-006-9199-3 17191139

[B31] Grammas P. (2011). Neurovascular dysfunction, inflammation and endothelial activation: implications for the pathogenesis of Alzheimer’s disease. *J. Neuroinflam.* 8:26. 10.1186/1742-2094-8-26 21439035 PMC3072921

[B32] Grammas P. Ovase R. (2001). Inflammatory factors are elevated in brain microvessels in Alzheimer’s disease. *Neurobiol. Aging* 22 837–842. 10.1016/S0197-4580(01)00276-7 11754990

[B33] Grammas P. Reimann-Philipp U. Weigel P. H. (2000). Cerebrovasculature-mediated Neuronal Cell Death. *Ann. N. Y. Acad. Sci.* 903 55–60. 10.1111/J.1749-6632.2000.TB06350.X 10818489

[B34] Grammas P. Samany P. Thirumangalakudi L. (2006). Thrombin and inflammatory proteins are elevated in Alzheimer’s disease microvessels: implications for disease pathogenesis. *J. Alzheimers. Dis.* 9 51–58. 10.3233/JAD-2006-9105 16627934

[B35] Haiyuan L. Xue X. Min L. Lingyu W. Xianlin G. Hancong S. (2020). Study of quantity and function of endothelial progenitor cells in peripheral blood of patients with Alzheimer’s disease. *J. New Med.* 51:590. 10.3969/J.ISSN.0253-9802.2020.08.004

[B36] Halliday M. R. Pomara N. Sagare A. P. Mack W. J. Frangione B. Zlokovic B. V. (2013). Relationship between cyclophilin A levels and matrix metalloproteinase 9 activity in cerebrospinal fluid of cognitively normal apolipoprotein E4 carriers and blood-brain barrier breakdown. *JAMA Neurol.* 70 1198–1200. 10.1001/jamaneurol.2013.3841 24030206 PMC4047029

[B37] Halliday M. R. Rege S. V. Ma Q. Zhao Z. Miller C. A. Winkler E. A. (2016). Accelerated pericyte degeneration and blood-brain barrier breakdown in apolipoprotein E4 carriers with Alzheimer’s disease. *J. Cereb. Blood Flow Metab.* 36 216–227. 10.1038/jcbfm.2015.44 25757756 PMC4758554

[B38] Hampel H. Mesulam M. M. Cuello A. C. Farlow M. R. Giacobini E. Grossberg G. T. (2018). The cholinergic system in the pathophysiology and treatment of Alzheimer’s disease. *Brain* 141:1917. 10.1093/BRAIN/AWY132 29850777 PMC6022632

[B39] Hayek S. S. MacNamara J. Tahhan A. S. Awad M. Yadalam A. Ko Y. A. (2016). Circulating Progenitor Cells Identify Peripheral Arterial Disease in Patients with Coronary Artery Disease. *Circ. Res.* 119 564–571. 10.1161/CIRCRESAHA.116.308802 27267067 PMC4975640

[B40] Heiss C. Keymel S. Niesler U. Ziemann J. Kelm M. Kalka C. (2005). Impaired progenitor cell activity in age-related endothelial dysfunction. *J. Am. Coll. Cardiol.* 45 1441–1448. 10.1016/J.JACC.2004.12.074 15862416

[B41] Heller L. Thinard R. Chevalier M. Arpag S. Jing Y. Greferath R. (2020). Secretion of proteins and antibody fragments from transiently transfected endothelial progenitor cells. *J. Cell. Mol. Med.* 24 8772–8778. 10.1111/JCMM.15511 32610368 PMC7412409

[B42] Hill J. M. Zalos G. Halcox J. P. J. Schenke W. H. Waclawiw M. A. Quyyumi A. A. (2009). Circulating Endothelial Progenitor Cells, Vascular Function, and Cardiovascular Risk. *N. Engl. J. Med.* 348 593–600. 10.1056/NEJMOA022287 12584367

[B43] Hristov M. Erl W. Weber P. (2003). Endothelial progenitor cells: isolation and characterization. *Trends Cardiovasc. Med.* 13 201–206. 10.1016/S1050-1738(03)00077-X 12837583

[B44] Huang X. T. Zhang Y. Q. Li S. J. Li S. H. Tang Q. Wang Z. T. (2013). Intracerebroventricular Transplantation of Ex Vivo Expanded Endothelial Colony-Forming Cells Restores Blood–Brain Barrier Integrity and Promotes Angiogenesis of Mice with Traumatic Brain Injury. *J. Neurotrauma.* 30 2080–2088. https://home.liebertpub.com/neu 23957220 10.1089/neu.2013.2996 PMC3868401

[B45] Hur J. Yoon C. H. Kim H. S. Choi J. H. Kang H. J. Hwang K. K. (2004). Characterization of Two Types of Endothelial Progenitor Cells and Their Different Contributions to Neovasculogenesis. *Arterioscler. Thromb. Vasc. Biol.* 24 288–293. 10.1161/01.ATV.0000114236.77009.06 14699017

[B46] Hussain B. Fang C. Chang J. (2021). Blood-Brain Barrier Breakdown: an Emerging Biomarker of Cognitive Impairment in Normal Aging and Dementia. *Front. Neurosci.* 15:688090. 10.3389/FNINS.2021.688090 34489623 PMC8418300

[B47] International A. D. (2019). *World Alzheimer Report 2019: attitudes to Dementia.* Available online at: https://www.alzint.org/resource/world-alzheimer-report-2019/ [Accessed October 20, 2021]

[B48] Ito M. T. da Silva Costa S. M. Baptista L. C. Carvalho-Siqueira G. Q. Albuquerque D. M. Rios V. M. (2020). Angiogenesis-Related Genes in Endothelial Progenitor Cells May Be Involved in Sickle Cell Stroke. *J. Am. Heart Assoc.* 9:e014143. 10.1161/JAHA.119.014143 32009522 PMC7033889

[B49] Jie K. E. Goossens M. H. J. van Oostrom O. Lilien M. R. Verhaar M. C. (2009). Circulating endothelial progenitor cell levels are higher during childhood than in adult life. *Atherosclerosis* 202 345–347. 10.1016/J.ATHEROSCLEROSIS.2008.05.012 18571177

[B50] Kapoor A. Gaubert A. Marshall A. Meier I. B. Yew B. Ho J. K. (2021). Increased levels of circulating angiogenic cells and signaling proteins in older adults with cerebral small vessel disease. *Front. Aging Neurosci.* 0:495. 10.3389/FNAGI.2021.711784 34650423 PMC8510558

[B51] Khoonsari P. E. Shevchenko G. Herman S. Remnestal J. Giedraitis V. Brundin R. (2019). Improved differential diagnosis of Alzheimer’s disease by integrating elisa and mass spectrometry-based cerebrospinal fluid biomarkers. *J. Alzheimers Dis.* 67 639–651. 10.3233/JAD-180855 30614806 PMC6398544

[B52] Koike M. A. Green K. N. Blurton-Jones M. LaFerla F. M. (2010). Oligemic hypoperfusion differentially affects tau and amyloid-β. *Am. J. Pathol.* 177 300–310. 10.2353/ajpath.2010.090750 20472896 PMC2893673

[B53] Kong X. D. Zhang Y. Liu L. Sun N. Zhang M. Y. Zhang J. N. (2011). Endothelial progenitor cells with Alzheimer’s disease. *Chin. Med. J.* 124 901–906. 10.3760/CMA.J.ISSN.0366-6999.2011.06.018 21518600

[B54] Kurz C. Walker L. Rauchmann B.-S. Perneczky R. (2021). Dysfunction of the blood-brain barrier in Alzheimer’s disease: evidence from human studies. *Neuropathol. Appl. Neurobiol.* 10.1111/NAN.12782 Epub online ahead of print. 34823269

[B55] Lee D. Rizer J. Selenica M. Reid P. Kraft C. Johnson A. (2010). LPS- induced inflammation exacerbates phospho-tau pathology in rTg4510 mice. *J. Neuroinflam.* 7:56. 10.1186/1742-2094-7-56 20846376 PMC2949628

[B56] Lee S. T. Chu K. Jung K. H. Jeon D. Bahn J. J. Kim J. H. (2010). Dysfunctional Characteristics of Circulating Angiogenic Cells in Alzheimer’s Disease. *J. Alzheimers Dis.* 19 1231–1240. 10.3233/JAD-2010-1315 20308789

[B57] Lee S. Chu K. Jung K. Park H. Kim D. Bahn J. (2009). Reduced circulating angiogenic cells in Alzheimer disease. *Neurology* 72 1858–1863. 10.1212/WNL.0B013E3181A711F4 19470969

[B58] Li L. Zhang X. Yang D. Luo G. Chen S. Le W. (2009). Hypoxia increases Abeta generation by altering beta- and gamma-cleavage of APP. *Neurobiol. Aging* 30 1091–1098. 10.1016/J.NEUROBIOLAGING.2007.10.011 18063223

[B59] Lin K. C. Chai H. T. Chen K. H. Sung P. H. Chiang J. Y. Shao P. L. (2020). Intra-carotid arterial transfusion of circulatory-derived autologous endothelial progenitor cells in rodent after ischemic stroke-evaluating the impact of therapeutic time points on prognostic outcomes. *Stem Cell Res. Ther.* 11 1–15. 10.1186/S13287-020-01739-Y/FIGURES/10 32503671 PMC7275327

[B60] Mahley R. W. (2016). Apolipoprotein E: from cardiovascular disease to neurodegenerative disorders. *J. Mol. Med.* 94:739. 10.1007/S00109-016-1427-Y 27277824 PMC4921111

[B61] Maler J. M. Spitzer P. Lewczuk P. Kornhuber J. Herrmann M. Wiltfang J. (2006). Decreased circulating CD34+ stem cells in early Alzheimer’s disease: evidence for a deficient hematopoietic brain support? *Mol. Psychiatry* 1112 1113–1115. 10.1038/sj.mp.4001913 17033629

[B62] Malinovskaya N. A. Komleva Y. K. Salmin V. V. Morgun A. V. Shuvaev A. N. Panina Y. A. (2016). Endothelial progenitor cells physiology and metabolic plasticity in brain angiogenesis and blood-brain barrier modeling. *Front. Physiol.* 7:599. 10.3389/fphys.2016.00599 27990124 PMC5130982

[B63] Mhatre M. Nguyen A. Kashani S. Pham T. Adesina A. Grammas P. (2004). Thrombin, a mediator of neurotoxicity and memory impairment. *Neurobiol. Aging* 25 783–793. 10.1016/J.NEUROBIOLAGING.2003.07.007 15165703

[B64] Miners J. S. Kehoe P. G. Love S. Zetterberg H. Blennow K. (2019). CSF evidence of pericyte damage in Alzheimer’s disease is associated with markers of blood-brain barrier dysfunction and disease pathology. *Alzheimers Res. Ther.* 11 1–6. 10.1186/S13195-019-0534-8/FIGURES/2 31521199 PMC6745071

[B65] Minoshima S. Giordani B. Berent S. Frey K. A. Foster N. L. Kuhl D. E. (1997). Metabolic reduction in the posterior cingulate cortex in very early Alzheimer’s disease. *Ann. Neurol.* 42 85–94. 10.1002/ANA.410420114 9225689

[B66] Moazzami K. Wittbrodt M. Lima B. Kim J. Hammadah M. Ko Y. (2020). Circulating Progenitor Cells and Cognitive Impairment in Men and Women with Coronary Artery Disease. *J. Alzheimers. Dis.* 74 659–668. 10.3233/JAD-191063 32083582 PMC8389168

[B67] Montagne A. Barnes S. R. Sweeney M. D. Halliday M. R. Sagare A. P. Zhao Z. (2015). Blood-Brain barrier breakdown in the aging human hippocampus. *Neuron* 85 296–302. 10.1016/j.neuron.2014.12.032 25611508 PMC4350773

[B68] Montagne A. Nation D. A. Sagare A. P. Barisano G. Sweeney M. D. Chakhoyan A. (2020). APOE4 leads to blood–brain barrier dysfunction predicting cognitive decline. *Nature* 581 71–76. 10.1038/s41586-020-2247-3 32376954 PMC7250000

[B69] Montagne A. Zhao Z. Zlokovic B. V. (2017). Alzheimer’s disease: a matter of blood-brain barrier dysfunction? *J. Exp. Med.* 214 3151–3169. 10.1084/JEM.20171406 29061693 PMC5679168

[B70] Mooradian A. Chung H. Shah G. (1997). GLUT-1 expression in the cerebra of patients with Alzheimer’s disease. *Neurobiol. Aging* 18 469–474. 10.1016/S0197-4580(97)00111-5 9390772

[B71] Mufson E. J. Counts S. E. Perez S. E. Ginsberg S. D. (2008). Cholinergic system during the progression of Alzheimer’s disease: therapeutic implications. *Expert Rev. Neurother.* 8:1703. 10.1586/14737175.8.11.1703 18986241 PMC2631573

[B72] Nation D. A. Sweeney M. D. Montagne A. Sagare A. P. D’Orazio L. M. Pachicano M. (2019). Blood–brain barrier breakdown is an early biomarker of human cognitive dysfunction. *Nat. Med.* 252 270–276. 10.1038/s41591-018-0297-y 30643288 PMC6367058

[B73] Nation D. A. Tan A. Dutt S. McIntosh E. C. Yew B. Ho J. K. (2018). Circulating Progenitor Cells Correlate with Memory, Posterior Cortical Thickness, and Hippocampal Perfusion. *J. Alzheimers Dis.* 61 91–101. 10.3233/JAD-170587 29103037 PMC5924766

[B74] Nelson A. R. A. Sweeney M. D. M. Sagare A. P. A. Zlokovic B. V. (2016). Neurovascular dysfunction and neurodegeneration in dementia and Alzheimer’s disease. *Biochim. Biophys. Acta - Mol. Basis Dis.* 1862 887–900. 10.1016/j.bbadis.2015.12.016 26705676 PMC4821735

[B75] Paris D. Townsend K. Quadros A. Humphrey J. Sun J. Brem S. (2004). Inhibition of angiogenesis by Aβ peptides. *Angiogenesis* 7 75–85. 10.1023/B:AGEN.0000037335.17717.bf 15302999

[B76] Paroni G. Bisceglia P. Seripa D. (2019). Understanding the Amyloid Hypothesis in Alzheimer’s Disease. *J. Alzheimers. Dis.* 68 493–510. 10.3233/JAD-180802 30883346

[B77] Paul J. Strickland S. Melchor J. (2007). Fibrin deposition accelerates neurovascular damage and neuroinflammation in mouse models of Alzheimer’s disease. *J. Exp. Med.* 204 1999–2008. 10.1084/JEM.20070304 17664291 PMC2118680

[B78] Pereira J. B. Janelidze S. Ossenkoppele R. Kvartsberg H. Brinkmalm A. Mattsson-Carlgren N. (2021). Untangling the association of amyloid-β and tau with synaptic and axonal loss in Alzheimer’s disease. *Brain* 144 310–324. 10.1093/BRAIN/AWAA395 33279949 PMC8210638

[B79] Pías-Peleteiro J. Campos F. Perez-Mato M. Lopez-Arias E. Rodriguez-Yanez M. Castillo J. (2017). Endothelial Progenitor Cells as a Therapeutic Approach for Intracerebral Hemorrhage. *Curr. Pharm. Des.* 23 2238–2251. 10.2174/1381612822666161221153937 28003010

[B80] Regan R. Guo Y. (1998). Toxic effect of hemoglobin on spinal cord neurons in culture. *J. Neurotrauma* 15 645–653. 10.1089/NEU.1998.15.645 9726263

[B81] Rodríguez C. Sobrino T. Agulla J. Bobo-Jiménez V. Ramos-Araque M. E. Duarte J. J. (2016). Neovascularization and functional recovery after intracerebral hemorrhage is conditioned by the Tp53 Arg72Pro single-nucleotide polymorphism. *Cell Death Differ.* 241 144–154. 10.1038/cdd.2016.109 27768124 PMC5260494

[B82] Rodríguez-Osorio X. Sobrino T. Brea D. Martínez F. Castillo J. Leira R. (2012). Endothelial progenitor cells: a new key for endothelial dysfunction in migraine. *Neurology* 79 474–479. 10.1212/WNL.0B013E31826170CE 22815557

[B83] Ryu J. K. McLarnon J. G. (2009). A leaky blood-brain barrier, fibrinogen infiltration and microglial reactivity in inflamed Alzheimer’s disease brain. *J. Cell. Mol. Med.* 13 2911–2925. 10.1111/j.1582-4934.2008.00434.x 18657226 PMC4498946

[B84] Safar M. M. Arab H. H. Rizk S. M. El-Maraghy S. A. (2016). Bone Marrow-Derived Endothelial Progenitor Cells Protect Against Scopolamine-Induced Alzheimer-Like Pathological Aberrations. *Mol. Neurobiol.* 53 1403–1418. 10.1007/s12035-014-9051-8 25526861

[B85] Sagare A. P. Bell R. D. Zlokovic B. V. (2012). Neurovascular dysfunction and faulty amyloid β-peptide clearance in Alzheimer disease. *Cold Spring Harb. Perspect. Med.* 2:a011452. 10.1101/cshperspect.a011452 23028132 PMC3475405

[B86] Salmon E. Sadzot B. Maquet P. Degueldre C. Lemaire C. Rigo P. (1994). Differential Diagnosis of Alzheimer’s Disease with PET. *J. Nucl. Med.* 35 391–398. 8113882

[B87] Sengillo J. D. Winkler E. A. Walker C. T. Sullivan J. S. Johnson M. Zlokovic B. V. (2013). Deficiency in mural vascular cells coincides with blood-brain barrier disruption in alzheimer’s disease. *Brain Pathol.* 23 303–310. 10.1111/bpa.12004 23126372 PMC3628957

[B88] Sidney L. E. Branch M. J. Dunphy S. E. Dua H. S. Hopkinson A. (2014). Concise Review: evidence for CD34 as a Common Marker for Diverse Progenitors. *Stem Cells* 32:1380. 10.1002/STEM.1661 24497003 PMC4260088

[B89] Sobrino T. Arias S. Pérez-Mato M. Agulla J. Brea D. Rodríguez-Yáñez M. (2011). Cd34+ progenitor cells likely are involved in the good functional recovery after intracerebral hemorrhage in humans. *J. Neurosci. Res.* 89 979–985. 10.1002/JNR.22627 21488087

[B90] Sobrino T. Blanco M. Pérez-Mato M. Rodríguez-Yáñez M. Castillo J. (2012a). Increased levels of circulating endothelial progenitor cells in patients with ischaemic stroke treated with statins during acute phase. *Eur. J. Neurol.* 19 1539–1546. 10.1111/J.1468-1331.2012.03770.X 22640405

[B91] Sobrino T. Pérez-Mato M. Brea D. Rodríguez-Yáñez M. Blanco M. Castillo J. (2012b). Temporal profile of molecular signatures associated with circulating endothelial progenitor cells in human ischemic stroke. *J. Neurosci. Res.* 90 1788–1793. 10.1002/JNR.23068 22513751

[B92] Sobrino T. Hurtado O. Moro M. Á Rodríguez-Yáñez M. Castellanos M. Brea D. (2007). The increase of circulating endothelial progenitor cells after acute ischemic stroke is associated with good outcome. *Stroke* 38 2759–2764. 10.1161/STROKEAHA.107.484386 17761925

[B93] Soto-Rojas L. O. Campa-Córdoba B. B. Harrington C. R. Salas-Casas A. Hernandes-Alejandro M. Villanueva-Fierro I. (2021). Insoluble Vascular Amyloid Deposits Trigger Disruption of the Neurovascular Unit in Alzheimer’s Disease Brains. *Int. J. Mol. Sci.* 22:3654. 10.3390/IJMS22073654 33915754 PMC8036769

[B94] Steinman J. Sun H. S. Feng Z. P. (2021). Microvascular Alterations in Alzheimer’s Disease. *Front. Cell. Neurosci.* 14:472. 10.3389/FNCEL.2020.618986/BIBTEX PMC7849053 33536876

[B95] Stellos K. Panagiota V. Sachsenmaier S. Trunk T. Straten G. Leyhe T. (2010). Increased circulating progenitor cells in Alzheimer’s disease patients with moderate to severe dementia: evidence for vascular repair and tissue regeneration? *J. Alzheimers Dis.* 19 591–600. 10.3233/JAD-2010-1261 20110604

[B96] Storck S. E. Hartz A. M. S. Bernard J. Wolf A. Kachlmeier A. Mahringer A. (2018). The concerted amyloid-beta clearance of LRP1 and ABCB1/P-gp across the blood-brain barrier is linked by PICALM. *Brain. Behav. Immun.* 73 21–33. 10.1016/J.BBI.2018.07.017 30041013 PMC7748946

[B97] Sweeney M. D. Sagare A. P. Zlokovic B. V. (2018). Blood–brain barrier breakdown in Alzheimer’s disease and other neurodegenerative disorders. *Nat. Rev. Neurol.* 14:133. 10.1038/NRNEUROL.2017.188 29377008 PMC5829048

[B98] Sweeney M. D. Zhao Z. Montagne A. Nelson A. R. Zlokovic B. V. (2019). Blood-Brain Barrier: from Physiology to Disease and Back. *Physiol. Rev.* 99:21. 10.1152/PHYSREV.00050.2017 30280653 PMC6335099

[B99] Tao Q. Ang T. F. A. DeCarli C. Auerbach S. H. Devine S. Stein T. D. (2018). Association of Chronic Low-grade Inflammation With Risk of Alzheimer Disease in ApoE4 Carriers. *JAMA Netw. Open* 1:e183597. 10.1001/JAMANETWORKOPEN.2018.3597 30646251 PMC6324596

[B100] Theofilis P. Sagris M. Oikonomou E. Antonopoulos A. S. Siasos G. Tsioufis C. (2021). Inflammatory Mechanisms Contributing to Endothelial Dysfunction. *Biomedicines* 9:781. 10.3390/BIOMEDICINES9070781 34356845 PMC8301477

[B101] Thirumangalakudi L. Samany P. Owoso A. Wiskar B. Grammas P. (2006). Angiogenic proteins are expressed by brain blood vessels in Alzheimer’s disease. *J. Alzheimers. Dis.* 10 111–118. 10.3233/JAD-2006-10114 16988487

[B102] Tian H. Davidowitz E. Lopez P. Emadi S. Moe J. Sierks M. (2013). Trimeric tau is toxic to human neuronal cells at low nanomolar concentrations. *Int. J. Cell Biol.* 2013:260787. 10.1155/2013/260787 24159335 PMC3789488

[B103] Uetani H. Hirai T. Hashimoto M. Ikeda M. Kitajima M. Sakamoto F. (2013). Prevalence and topography of small hypointense foci suggesting microbleeds on 3T susceptibility-weighted imaging in various types of dementia. *Am. J. Neuroradiol.* 34 984–989. 10.3174/ajnr.A3332 23124636 PMC7964666

[B104] Van Assema D. M. E. Lubberink M. Bauer M. Van Der Flier W. M. Schuit R. C. Windhorst A. D. (2012). Blood-brain barrier P-glycoprotein function in Alzheimer’s disease. *Brain* 135 181–189. 10.1093/brain/awr298 22120145

[B105] Van De Haar H. J. Burgmans S. Jansen J. F. A. Van Osch M. J. P. Van Buchem M. A. Muller M. (2016a). Blood-brain barrier leakage in patients with early Alzheimer disease. *Radiology* 281 527–535. 10.1148/radiol.2016152244 27243267

[B106] Van De Haar H. J. Jansen J. Mjp van O. van Buchem M. Muller M. Wong S. (2016b). Neurovascular unit impairment in early Alzheimer’s disease measured with magnetic resonance imaging. *Neurobiol. Aging* 45 190–196. 10.1016/J.NEUROBIOLAGING.2016.06.006 27459939

[B107] Wisniewski H. M. Kozlowski P. B. (1982). Evidence for Blood-Brain Barrier Changes in Senile Dementia of the Alzheimer Type (SDAT). *Ann. N. Y. Acad. Sci.* 396 119–129. 10.1111/J.1749-6632.1982.TB26848.X 6185032

[B108] Wolz R. Julkunen V. Koikkalainen J. Niskanen E. Zhang D. Rueckert D. (2011). Multi-method analysis of MRI images in early diagnostics of Alzheimer’s disease. *PLoS One* 6:e25446. 10.1371/JOURNAL.PONE.0025446 22022397 PMC3192759

[B109] Wu Z. Guo H. Chow N. Sallstrom J. Bell R. Deane R. (2005). Role of the MEOX2 homeobox gene in neurovascular dysfunction in Alzheimer disease. *Nat. Med.* 11 959–965. 10.1038/NM1287 16116430

[B110] Xia S. Tai X. Wang Y. An X. Qian G. Dong J. (2012). Involvement of Gax Gene in Hypoxia-Induced Pulmonary Hypertension, Proliferation, and Apoptosis of Arterial Smooth Muscle Cells. *Am. J. Respir. Cell Mol. Biol.* 44. 66–73. 10.1165/RCMB.2008-0442OC 20160044

[B111] Xia W. H. Yang Z. Xu S. Y. Chen L. Zhang X. Y. Li J. (2012). Age-related decline in reendothelialization capacity of human endothelial progenitor cells is restored by shear stress. *Hypertension* 59 1225–1231. 10.1161/HYPERTENSIONAHA.111.179820 22547440

[B112] Yates P. A. Desmond P. M. Phal P. M. Steward C. Szoeke C. Salvado O. (2014). Incidence of cerebral microbleeds in preclinical Alzheimer disease. *Neurology* 82 1266–1273. 10.1212/WNL.0000000000000285 24623839 PMC4001205

[B113] Yoder M. C. (2012). Human endothelial progenitor cells. *Cold Spring Harb. Perspect. Med.* 2:a006692. 10.1101/cshperspect.a006692 22762017 PMC3385946

[B114] Yuan X. Mei B. Zhang L. Zhang C. Zheng M. Liang H. (2016). Enhanced penetration of exogenous EPCs into brains of APP/PS1 transgenic mice. *Am. J. Transl. Res.* 8 1460–1470. 27186272 PMC4859631

[B115] Zenaro E. Pietronigro E. Bianca V. Della Piacentino G. Marongiu L. Budui S. (2015). Neutrophils promote Alzheimer’s disease-like pathology and cognitive decline via LFA-1 integrin. *Nat. Med.* 21 880–886. 10.1038/nm.3913 26214837

[B116] Zhang S. Zhi Y. Li F. Huang S. Gao H. Han Z. (2018). Transplantation of in vitro cultured endothelial progenitor cells repairs the blood-brain barrier and improves cognitive function of APP/PS1 transgenic AD mice. *J. Neurol. Sci.* 387 6–15. 10.1016/J.JNS.2018.01.019 29571873

[B117] Zhang X. Zhou K. Wang R. Cui J. Lipton S. A. Liao F. F. (2007). Hypoxia-inducible factor 1α (HIF-1α)-mediated hypoxia increases BACE1 expression and β-amyloid generation. *J. Biol. Chem.* 282 10873–10880. 10.1074/jbc.M608856200 17303576

[B118] Zlokovic B. (2005). Neurovascular mechanisms of Alzheimer’s neurodegeneration. *Trends Neurosci.* 28 202–208. 10.1016/J.TINS.2005.02.001 15808355

[B119] Zlokovic B. (2011). Neurovascular pathways to neurodegeneration in Alzheimer’s disease and other disorders. *Nat. Rev. Neurosci.* 12 723–38. 10.1038/NRN3114 22048062 PMC4036520

